# Hypothalamic connectivities predict individual differences in ADT-elicited changes in working memory and quality of life in prostate cancer patients

**DOI:** 10.1038/s41598-022-13361-4

**Published:** 2022-06-10

**Authors:** Shefali Chaudhary, Alicia Roy, Christine Summers, Simon Zhornitsky, Tim Ahles, Chiang-Shan R. Li, Herta H. Chao

**Affiliations:** 1 Department of Psychiatry, Yale University School of Medicine, CMHC S110, 34 Park Street, New Haven, CT 06519 USA; 2 Cancer Center, VA Connecticut Healthcare System, 950 Campbell Avenue, West Haven, CT 06516 USA; 3 Department of Psychiatry and Behavioral Sciences, Memorial Sloan Kettering Cancer Center, New York, NY USA; 4 Department of Psychiatry, Yale University, New Haven, CT 06520 USA; 5 Department of Neuroscience, Yale University, New Haven, CT 06520 USA; 6 Interdepartmental Neuroscience Program, Yale University School of Medicine, Yale University, New Haven, CT 06520 USA; 7 Wu Tsai Institute, Yale University, New Haven, CT 06520 USA; 8 Department of Medicine and Yale Comprehensive Cancer Center, Yale University School of Medicine, New Haven, CT 06519 USA

**Keywords:** Urological cancer, Biomarkers, Neurology, Oncology

## Abstract

Androgen deprivation therapy (ADT) has been associated with adverse effects on cognition. However, we currently lack understanding of the neurobiology and prognostic markers of these effects. Given that ADT acts via the hypothalamus–pituitary–gonadal axis, we assessed whether baseline hypothalamic resting state functional connectivity (rsFC) could predict changes in working memory and quality of life in prostate cancer patients following androgen deprivation. In a prospective observational study, 28 men with non-metastatic prostate cancer receiving ADT and 38 patients not receiving ADT (controls), matched in age, years of education and Montreal Cognitive Assessment score, participated in brain imaging at baseline, and N-back task and quality-of-life (QoL) assessments at baseline and at 6 months follow-up. Imaging data were processed with published routines and evaluated at a corrected threshold. ADT and control groups did not differ in N-back performance or QoL across time points. In ADT, the changes in 0-back correct response rate (follow-up—baseline) were correlated with baseline hypothalamus-precentral gyrus rsFC; the changes in 1-back correct response rate and reaction time were each correlated with hypothalamus-middle frontal gyrus and superior parietal lobule rsFC. The changes in physical well-being subscore of QoL were correlated with baseline hypothalamus-anterior cingulate and cuneus rsFC. The hypothalamus rsFCs predicted N-back and QoL change with an area under the receiver operating characteristic curve of 0.93 and 0.73, respectively. Baseline hypothalamus-frontoparietal and salience network rsFC’s predict inter-subject variations in the changes in working-memory and QoL following 6 months of ADT. Whether and how hypothalamic rsFCs may predict the cognitive and QoL effects with longer-term ADT remain to be investigated.

## Introduction

Androgen deprivation therapy (ADT) is the standard primary treatment for men with prostate cancer^1^. Despite the potential survival benefits, ADT is accompanied with physiological side effects that may compromise patient’s quality of life (QoL)^1,2^. Further, testosterone affects cognition possibly via modulation of neuronal signaling and connectivity, and plays a role in limiting the production of β-amyloid peptide and preventing *N*-methyl-d-aspartate excitotoxicity^1,3^. ADT results in reduction in the level of bioavailable testosterone and may negatively affect cognition^4^. However, according to a recent review, testosterone levels alone did not appear to predict cognitive decline or development of Alzheimer’s disease, nor does testosterone replacement therapy significantly affect cognition^5^ in elderly men. Similarly, in prostate cancer patients, the neurobiology of ADT-associated cognitive changes are not fully understood, and the predictors of such changes have yet been investigated systematically^3,6^.

Resting-state functional connectivity (rsFC) characterizes intrinsic functional organization of the brain and has successfully predicted changes in cognitive functions during healthy aging and in neurological illnesses^7–10^. Studies have noted the contribution of sex steroids to modulation of brain functional connectivity^11^. In support, prostate cancer patients with and without ADT demonstrate different patterns of regional connectivities. For instance, a recent study showed enhanced rsFC in regions with high androgen receptor expression in prostate cancer patients undergoing ADT, as compared to those who did not receive ADT^12^. Although a direct correlation between connectivity metrics and cognition was not reported, cognitive performance was worse in ADT patients and the changes in rsFC may reflect impaired brain functionality^12^. Importantly, in mild cognitive impairment, baseline rsFC predicted later development of dementia and Alzheimer disease with 80–90% accuracy^13,14^. RsFC also predicted treatment responses in patients with neuropsychiatric disorders^15–18^, suggesting its utility as a prognostic marker of clinical outcomes.

In the present study, we assessed whether baseline hypothalamic rsFC could predict changes in working memory and quality of life in prostate cancer patients at 6 months following androgen deprivation. ADT acts via the hypothalamus–pituitary–gonadal (HPG) axis and achieves its therapeutic effects^19^ by lowering the levels of androgen, which plays a critical role in driving proliferation of prostate cancer possibly via inducing cell cycle growth and blocking apoptosis^20^. Hypothalamus secretes gonadotropin-releasing hormone (GnRH) in a pulsatile manner, which stimulates the release of luteinizing hormone (LH)^21^. ADT with GnRH agonists initially stimulates this pathway, resulting in enhanced release of LH and androgens in men^22–24^. With continued administration, GnRH receptors on the pituitary become desensitized, and the levels of gonadotropin and androgen decline, thereby inhibiting tumor growth^23,25^. Thus, ADT with GnRH agonists achieves its therapeutic effects by acting directly on the hypothalamus.

In accord, animal studies have extensively documented the effects of androgen deprivation on hypothalamic structures and functions^26–28^. Importantly, hypothalamic circuits regulate object memory formation^29^ and play an instrumental role in motivation, arousal, and affect processing^30,31^. A meta-analysis reported HPG dysregulation, as evidenced in high night-time cortisol level as well as diurnal drop and awakening response, as a potential predictor of cognitive impairment in older individuals^32^. Another meta-analysis reported that variants of HPG axis-related genes, including those for corticotropin releasing hormone (CRH), CRH-binding protein, CRH-receptor, glucocorticoid and mineralocorticoid receptors, may differentially affect cognitive performance^33^. Global dysfunction of the HPG axis has often been described in the pathophysiology of Alzheimer’s disease^34,35^. Further, hypothalamic neuronal activities conduce to learning and memory independently of motivation, arousal, and anxiety^36–38^. A recent study associated N-back working memory performance and HPG axis response in healthy young adults^39^. Another study noted reduced hypothalamus-middle frontal rsFC in link with diminished attention in sleep-deprived men^40^. Further, we previously reported altered hypothalamus-precentral gyrus rsFC in association with 0-back correct response rate in prostate cancer patients on ADT^41^.

A review of longitudinal studies in humans and animals showed working memory, visuospatial memory, and executive functioning to be the cognitive domains most affected by androgen deprivation^1^. However, a meta-analysis noted effects of ADT on visuomotor performance but not working memory^42^. Another study reported worse visuospatial function and visual memory but overall intact cognitive performance in prostate cancer patients on ADT relative to controls in a longitudinal setting^43^. On the other hand, despite the less-than-consistent findings, studies have reported substantial inter-subject variation in cognitive performance, as also noted in women with breast cancer who received chemotherapy^44–47^. In particular, no studies to our knowledge have aimed to identify the neural predictors of individual variation in the impact of ADT on cognition.

Here, we evaluated hypothalamus rsFC as a predictor of ADT-associated changes in working memory and quality of life. We hypothesized that hypothalamus rsFC would predict individual variation in accuracy and reaction time in an N-back task and quality of life in prostate cancer patients receiving ADT.

## Materials and methods

### Participants and clinical profiles

Patient recruitment criteria and procedures followed our earlier studies^2,41,48^. Patients 55–75 years of age and with biopsy-proven prostate adenocarcinoma without distant metastases were recruited from the Medical Oncology and Urology Clinics at the West Haven VA Connecticut Healthcare System. Following current National Comprehensive Cancer Network and American Urological Society practice guidelines, treatments were not affected by patients’ decision of participation in the study. All patients prescribed ADT as adjuvant treatment or due to biochemical recurrence were contacted for participation. ADT consisted of medical castration with an LH-RH agonist (Goserelin or Leuprolide) subcutaneously for 6 months, after a lead-in period with Bicalutamide 50 mg daily. Patients with non-metastatic prostate cancer who had never been treated with ADT participated as controls (CON). For both ADT and CON, exclusion criteria were: Eastern Cooperative Oncology Group Performance Status > 1; active second malignancy; significant cardiovascular, liver, renal, or neurological disease; use of any investigational drugs or contraindications, including claustrophobia, for magnetic resonance imaging (MRI); current substance (except nicotine) use disorders (use of illicit substances were verified by a urine test); history of Axis I psychiatric illness; history of traumatic brain injury or concussions causing loss of consciousness. All participants underwent a health questionnaire interview to ensure eligibility for MRI. Participants who had a prostatectomy were at least 3 months from their surgery and had fully recovered from anesthesia before study entry. Participants who were to receive radiation to the prostate underwent baseline assessment and MR scan before starting any treatment and had to be fully recovered from any acute side effects of radiation at the time of their follow-up assessments. In addition to measuring serum testosterone and prostate-specific antigen as part of their routine bloodwork at every assessment, all participants underwent determination of other hormonal (e.g., cortisol and thyroid hormone) levels that could potentially affect cognitive function.

Among 100 candidates with non-metastatic prostate cancer, 78 who had never been treated with ADT were enrolled in the study. Thirty-six patients were scheduled for ADT (ADT group) and 42 patients served as controls (CON group). Thirty ADT and 40 CON completed both baseline (clinical assessment and MRI) and follow-up (clinical) assessments. However, 2 ADT and 2 CON were excluded due to excessive head movements during MR scans. Thus, the data from 28 ADT and 38 CON were included in the analyses (Supplementary Fig. S1).

### Study procedures and assessment of working memory and quality of life

All participants underwent clinical, including quality of life (QoL), and cognitive (N-back task) assessment at baseline and at 6-month follow-up. At baseline, participants were also assessed for global cognition using Montreal Cognitive Assessment (MoCA) and underwent MR imaging.

N-back task is a widely used paradigm to assess working memory, a form of short-term memory that provides temporary storage and manipulation of information necessary for complex cognitive tasks^49^ (Supplementary Fig. S2). Briefly, 0-, 1-, and 2-back trials were included in the N-back task, each imposing increasingly higher demand on working memory. We used the correct response rate (CR) and reaction time (RT) as outcome measures of N-back performance.

As a general measure of QoL, participants completed the Functional Assessment of Cancer Therapy-Prostate (FACT-P) questionnaire at baseline and at 6-month follow-up^50,51^. The cumulative score of FACT-P subscale scores: physical well-being (PWB), social well-being (SWB), emotional well-being (EWB), functional well-being (FWB), and prostate cancer subscale (PCS) formed the total QoL scores.

### Imaging protocol and data processing

Details of imaging data acquisition, processing, and analyses are described in the Supplement and follow our earlier study^41^. Briefly, subjects were scanned at baseline with a 3-Tesla Siemens Prisma system using a protocol as in our previous studies^41^. We pre-processed the imaging data and computed whole brain resting state functional connectivity (rsFC) of the hypothalamus for individual participants, following published routines in Statistical Parametric Mapping (SPM12)^31,52^.

Nuisance signals unlikely to reflect neural activity were removed using linear regression with the six head motion parameters from realignment, signals of the whole brain, ventricular system, white matter, and their first-order derivatives as covariates^31,53^. Images were checked for micro-head motions, followed by “scrubbing” to remove time points affected by head motions^54^ or DVARS(t) > 75^55^. We applied a temporal band-pass filter (0.009 Hz < f < 0.08 Hz) to the time course to obtain low-frequency fluctuations and computed the correlation maps to estimate rsFC of the hypothalamus^56,57^. We employed the hypothalamus mask from the WFU Pick-Atlas^58^ as the seed, as in an earlier study^52^. The correlation coefficients between the averaged time course of the hypothalamus seed and time courses of all other brain voxels were computed for each participant. Next, the correlation maps were converted into z-score maps by Fisher’s Z transform: z = 0.5log_e_ [1 + r/1 − r] (r = correlation coefficient) to obtain normally distributed maps^41^.

### Statistical analyses of clinical, behavioral, and imaging data

All statistical analyses of clinical and behavioral data were conducted with Stata (Stata Corp LLC, Texas, USA). We used linear mixed model via restricted maximum likelihood (REML) with random intercept across subjects to assess changes during follow-up from baseline in longitudinal variables, with group (ADT vs. CON) as a between-subject and time-point (follow-up vs. baseline) as a within-subject variable (fixed effects in model). In post-hoc analyses, between and within group differences were assessed using two- and paired-sample t-test, respectively. The results that met p < 0.05 (two-tailed) were considered statistically significant.

We employed SPM12 in group statistics of rsFC data. To examine hypothalamus rsFCs that could predict changes (follow-up vs. baseline) in N-back and QoL scores, we assessed the correlations between baseline hypothalamus rsFC and changes in N-back and QoL scores in ADT, using multiple regression module of SPM with baseline age, education, and MoCA scores as covariates. Clusters with hypothalamus rsFCs that met cluster p < 0.05, family-wise error (FWE)-corrected with a cluster-forming voxel p < 0.001, uncorrected were reported in MNI coordinates. We extracted cluster parameter (β) estimates for graphical presentation and further statistical analyses.

In an additional set of analyses, we targeted subprocesses of memory by contrasting the metrics of N-back performance across block conditions. For both CR and RT, we extracted these parameters: 2-back minus 0-back; 1-back minus 0-back; and 2-back minus 1-back, each representing maintenance load (2- vs. 0-item), replacement (1-step vs. no replacement), and shifting (2- shift vs. 1-step shift) subprocess of memory^59^. Replacement and shifting are components of updating. We repeated multiple regression, as described above, with these derived scores and presented the results in the Supplement.

### Prediction of change for worse vs. no-worse in N-back performance and QoL in ADT

We assessed how well hypothalamus rsFCs at baseline could predict inter-subject variation in the changes (follow-up—baseline) in N-back performance metrics and QoL scores using logistic regression. Changes in N-back and QoL scores were coded as 0 (worse) and 1 (no-worse). A worse change was assessed as for a difference > 0.5 SD (decrease in CR and QoL, and increase in RT) during follow-up as compared to baseline^60^; otherwise, no-worse. As there were multiple N-back performance metrics, we performed latent class analysis (LCA) of all six N-back metrics, i.e., 0-, 1-, 2-back CR and 0-, 1-, 2-back RT, each coded in 0 and 1, to obtain representative classes of overall worse/no-worse performance. LCA returned two classes of overall changes in N-back performance, with 0/1 to indicate worse/no-worse performance during follow-up vs. baseline (score distribution shown in Supplementary Fig. S3). We applied logistic regression using LCA (for N-back) and QoL classes as the response variable, hypothalmaus rsFCs as predictor, and baseline age, education, and MoCA scores as covariates. Model prediction accuracy was represented by the area under the curve (AUC) in receiver operating characteristic analysis.

### Ethics approval and consent to participate

The study was approved by the Human Investigation Committee at both the West Haven VA and Yale University School of Medicine (Ref. No.: HIC#2000020501) and was conducted in accordance with Declaration of Helsinki. All participants provided a written informed consent prior to the study.

## Results

### Baseline clinical profiles of the participants

At baseline, ADT and CON patients were comparable in age, years of education, MoCA score, testosterone (t_64_ = 1.0, p = 0.309), and cortisol (t_64_ = 0.3, p = 0.798) levels (Table 1). In addition, we observed a significant treatment × time interaction in testosterone level, as expected of the effects of ADT, but not in the cortisol level (Table 1).

**Table 1:** Demographic and clinical characteristics of the patients.

	ADT (n = 28)		CON (n = 38)		(t_64_/F_1,64_, p-value)
Age (year)	66.9 ± 6.9		64.5 ± 8.2		1.3, 0.211
Education (year)	13.7 ± 3.6		14.6 ± 2.9		1.0, 0.308
MoCA score	25.1 ± 2.6		26.2 ± 2.5		1.7, 0.089
	B	F	B	F	
T level (ng/ml)	3.7 ± 1.6	0.17 ± 0.7	4.1 ± 1.7	3.6 ± 1.6	57.2, < 0.001
C level (µg/dl)	9.3 ± 3.8	10.2 ± 3.6	9.5 ± 2.7	9.6 ± 2.5	0.68, 0.408

*MoCA* Montreal Cognitive Assessment, *T* testosterone, *C* cortisol, *B* baseline; *F* follow-up. For T and C levels, the statistics (F_1,64_, p-value) correspond to treatment × time interaction, while for the other variables the statistics (t_64_, p-value) correspond to two-sample t-tests comparing ADT and CON at the baseline.

### N-back performance and quality of life scores

**Figure 1:**
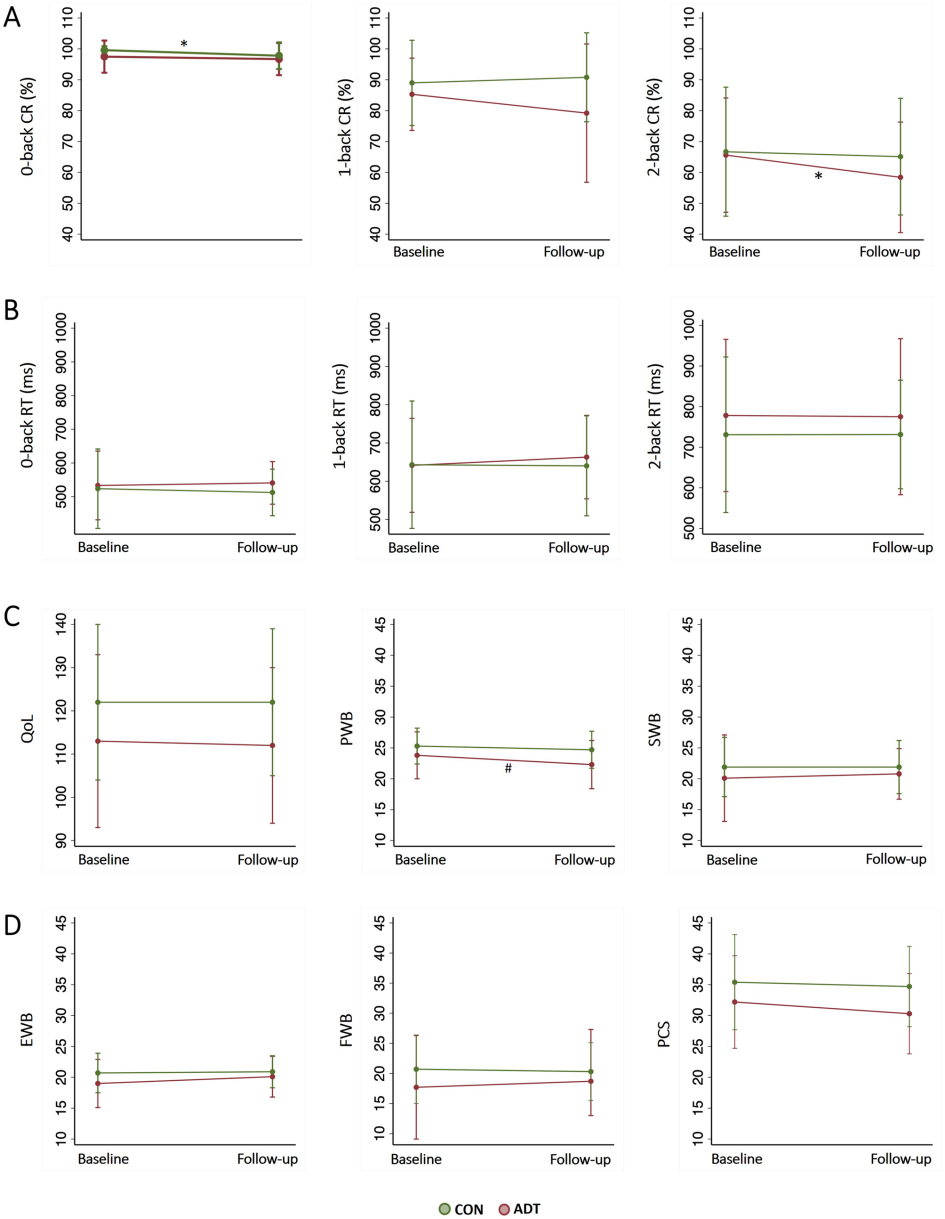
Distribution of (**A**) N-back correct response rates (%), (**B**) N-back response time (ms), (**C**) quality of life (QoL) score and sub-scores—physical well-being (PWB), subjective well-being (SWB), (**D**) emotional well-being (EWB), functional well-being (FWB)—and prostate cancer specific (PCS) score at baseline and follow-up in ADT (red) and CON (green). Data shown in group mean ± SD. *CR* correct response, *RT* reaction time. *p < 0.05, ^#^p = 0.078 in paired t-test.

In mixed model analyses, treatment (ADT/CON) × time (baseline/follow-up) interaction was not significant for any of the N-back performance metrics (Supplementary Table S1). Overall, mean correct response rate was higher and mean RT was lower in CON compared to ADT at both baseline and follow-up (Fig. 1, Supplementary Table S2). Addditionally, we observed a significant effect of treatment on 0-back and 1-back correct response rate, and a significant effect of time on 2-back correct response rate (Supplementary Table S1). Among the N-back metrics, baseline 0-back correct response rate (t_64_ = 2.4, p = 0.02) and follow-up 1-back correct response rate (t_64_ = 2.6, p = 0.01) were significantly higher in CON than in ADT. For other N-back metrics, baseline and follow-up measures were comparable between groups (p’s > 0.05).

Among memory subprocesses, correct response rate during replacement was better in CON compared to ADT during follow-up. Other baseline and follow-up comparisons were comparable between the two groups (Supplementary Table S3).

Treatment × time interaction was not significant for QoL scores and sub-scores (Supplementary Table S1). However, the mean scores were higher in CON than in ADT both during baseline and follow-up (Fig. 1, Supplementary Table S2). Significant main effect of treatment was noted for QoL, PWB and PCS scores; and significant main effect of time for PWB scores. The follow-up scores of QoL (t_64_ = 2.4, p = 0.019), PWB (t_64_ = 2.7, p = 0.008), and PCS (t_64_ = 2.7, p = 0.009) were lower in ADT than in CON. The other scores were not significantly different.

### Baseline hypothalamic rsFC predicting changes in N-back and QoL scores in ADT

**Figure 2:**
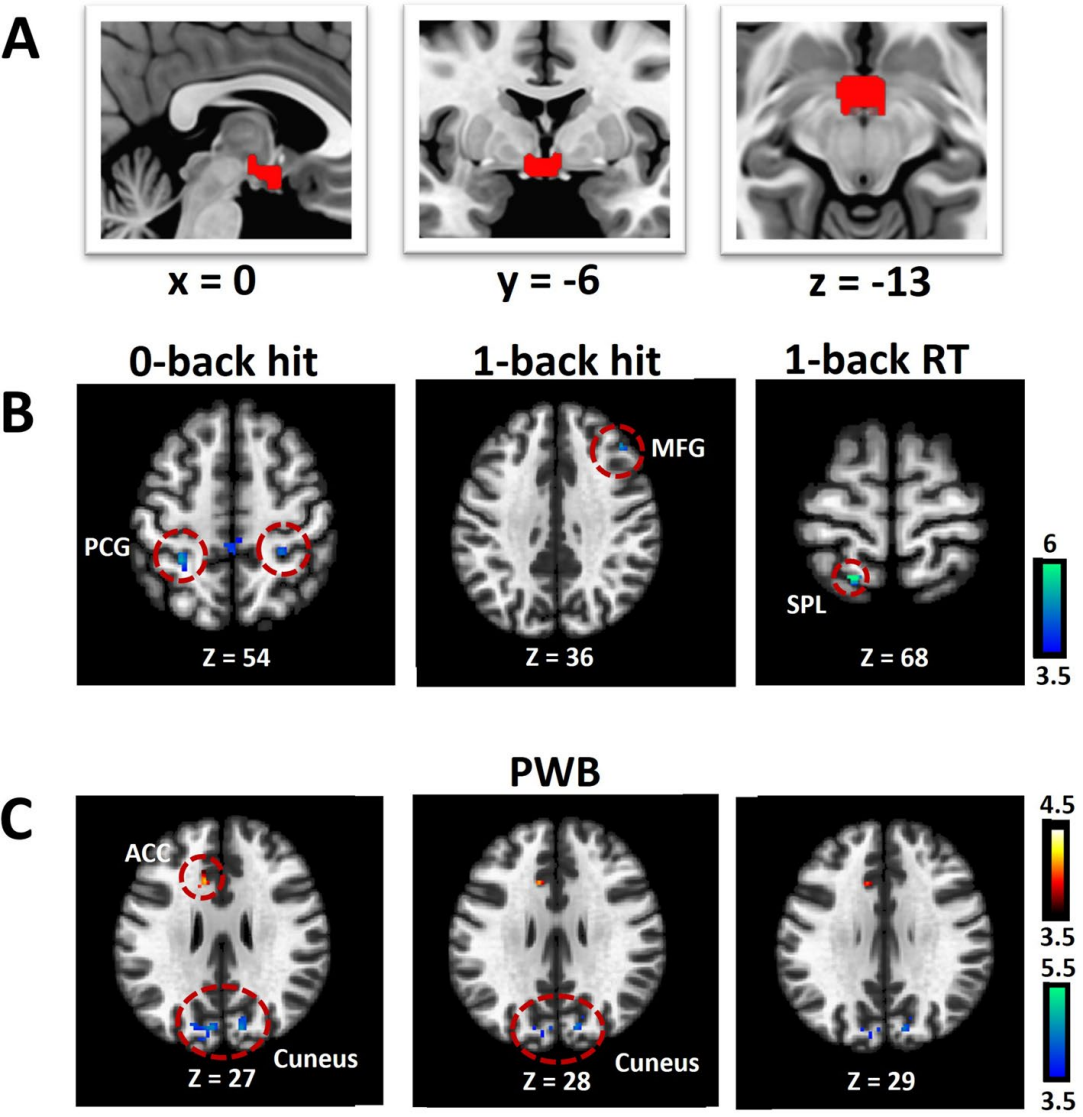
Hypothalamus (**A**: seed) rsFC correlates of change (follow-up versus baseline) in N-back scores (**B**) and QoL sub-score PWB (**C**) in ADT. The clusters were obtained using whole-brain regression with baseline age, education, MoCA as covariates, at cluster p < 0.05 FWE-corrected and cluster-forming voxel p < 0.001, uncorrected. Color bar shows voxel T-values. Warm/cool colors: positive/negative correlations. Hit: correct response rate (%), RT: reaction time (ms), PWB: physical well-being.

The hypothalamus seed is shown in Fig. 2A. We evaluated how baseline hypothalamus rsFC associated with changes (follow-up vs. baseline) in N-back and QoL scores for the whole-brain with baseline age, education and MoCA score as covariates at cluster p < 0.05 FWE-corrected with a cluster-forming voxel p < 0.001, uncorrected. In the ADT group, hypothalamus-bilateral precentral gyrus (PCG) rsFC was negatively correlated with change in 0-back correct response rate, hypothalamus-right middle frontal gyrus (MFG) rsFC was negatively correlated with change in 1-back correct response rate, and hypothalamus-left superior parietal lobule (SPL) rsFC was negatively correlated with change in 1-back RT (Fig. 2B).

Additionally, for memory subprocesses, the change in replacement RT, shift correct response rate and shift RT correlated negatively with hypothalamus-left SPL rsFC, positively with hypothalamus-left cerebellum rsFC, and negatively with hypothalamus-right cerebellum rsFC, respectively (Supplementary Fig. S4A). Regressions with the other measures did not reveal significant clusters.

Among the QoL scores, change in PWB was correlated positively with hypothalamus-left anterior cingulate cortex (ACC) rsFC and negatively with hypothalamus-bilateral cuneus rsFC (Fig. 2C). At the same threshold no clusters showed significant hypothalamus rsFCs in correlation with the changes in other QoL scores.

We also assessed whether baseline testosterone and cortisol levels could predict N-back and QoL changes in ADT. The results showed that baseline testosterone levels were not correlated with any of the N-back (all p’s > 0.102), and QoL (all p’s > 0.299) measures or hypothalamus rsFCs (all p’s > 0.164). Likewise, baseline cortisol levels were not correlated with any of the N-back (all p’s > 0.080), and QoL (p’s > 0.228) measures or hypothalamus rsFCs (all p’s > 0.170) across subjects.

**Figure 3:**
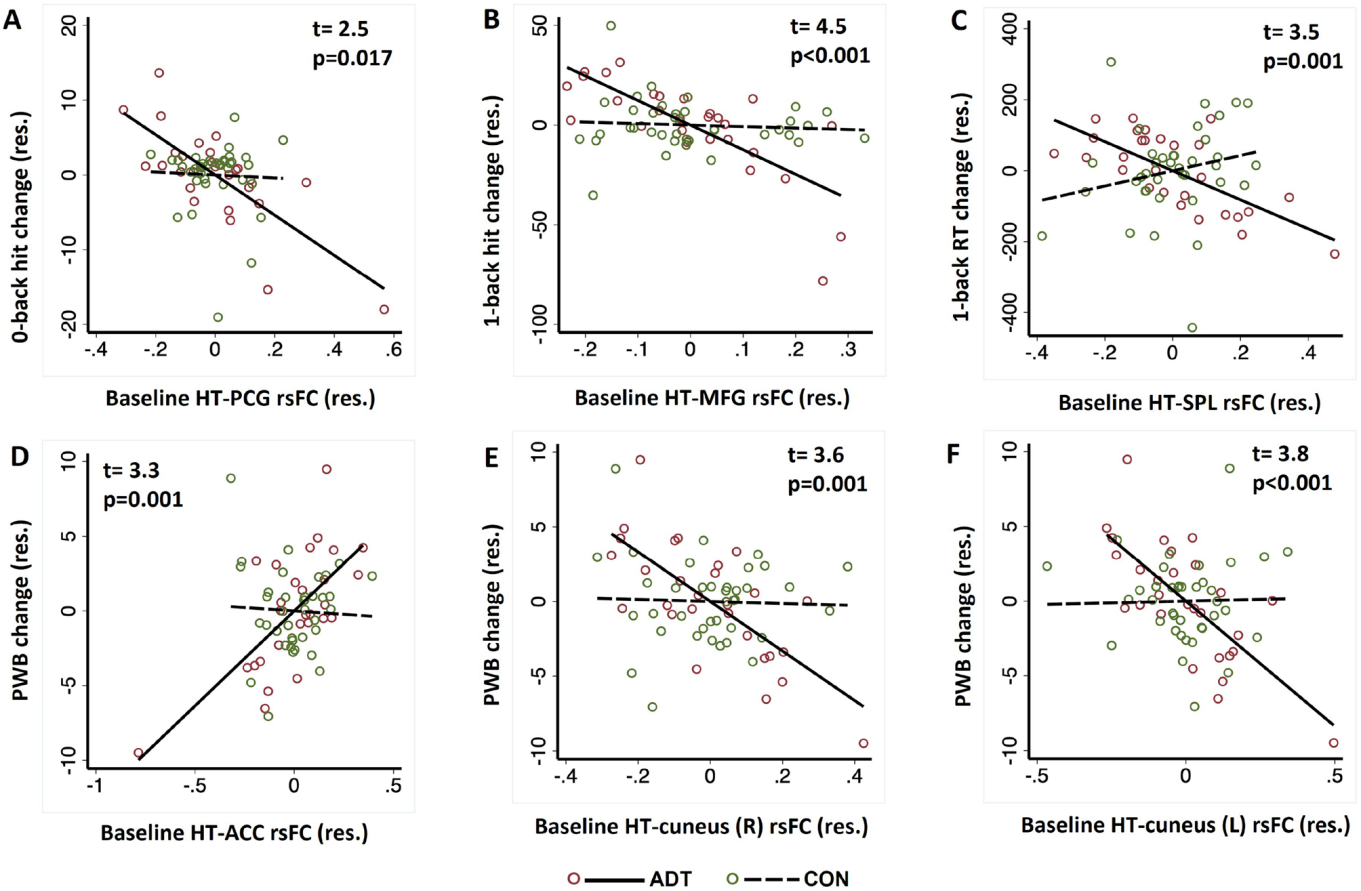
Pearson’s partial correlation between baseline hypothalamus (HT)—(**A**) bilateral precentral gyrus (PCG) rsFC and change (follow-up—baseline) in 0-back correct response rate (hit %); (**B**) right MFG rsFC and change in 1-back hit %; (**C**) left superior parietal lobule (SPL) rsFC and change in 1-back reaction time (RT); (**D**) left anterior cingulate cortex (ACC) rsFC and change in PWB; (**E**/**F**) right/left cuneus rsFC and change in PWB. Note that, because of the inclusion of covariates in the regression, the residuals are plotted here. The covariates included baseline age, years of education, and MoCA score. The insets show the t- and p-values of slope tests of ADT vs. CON in the regressions.

We computed the β estimtaes of hypothalamus rsFC of the regions identified in ADT for all participants (ADT and CON). Figure 3A–C show the correlations for ADT and CON separately and slope tests confirmed the differences in slope in the regressions of N-back metrics vs. rsFCs between groups. None of the regressions were significant in CON. Supplementary Figure S4B–D show the correlations with memory subprocesses (N-back derived metrics) separately in ADT and CON. Likewise, we computed the β estimtaes of hypothalamus rsFC with ACC and bilateral cuneus for all participants. Figure 3D–F show the correlations for ADT and CON separately and slope tests confirmed the differences in slope in the regressions of PWB score vs. rsFCs between groups.

### Accuracy of baseline hypothalamic rsFC in predicting worse vs. no-worse grouping in N-back and QoL scores in ADT

**Figure 4:**
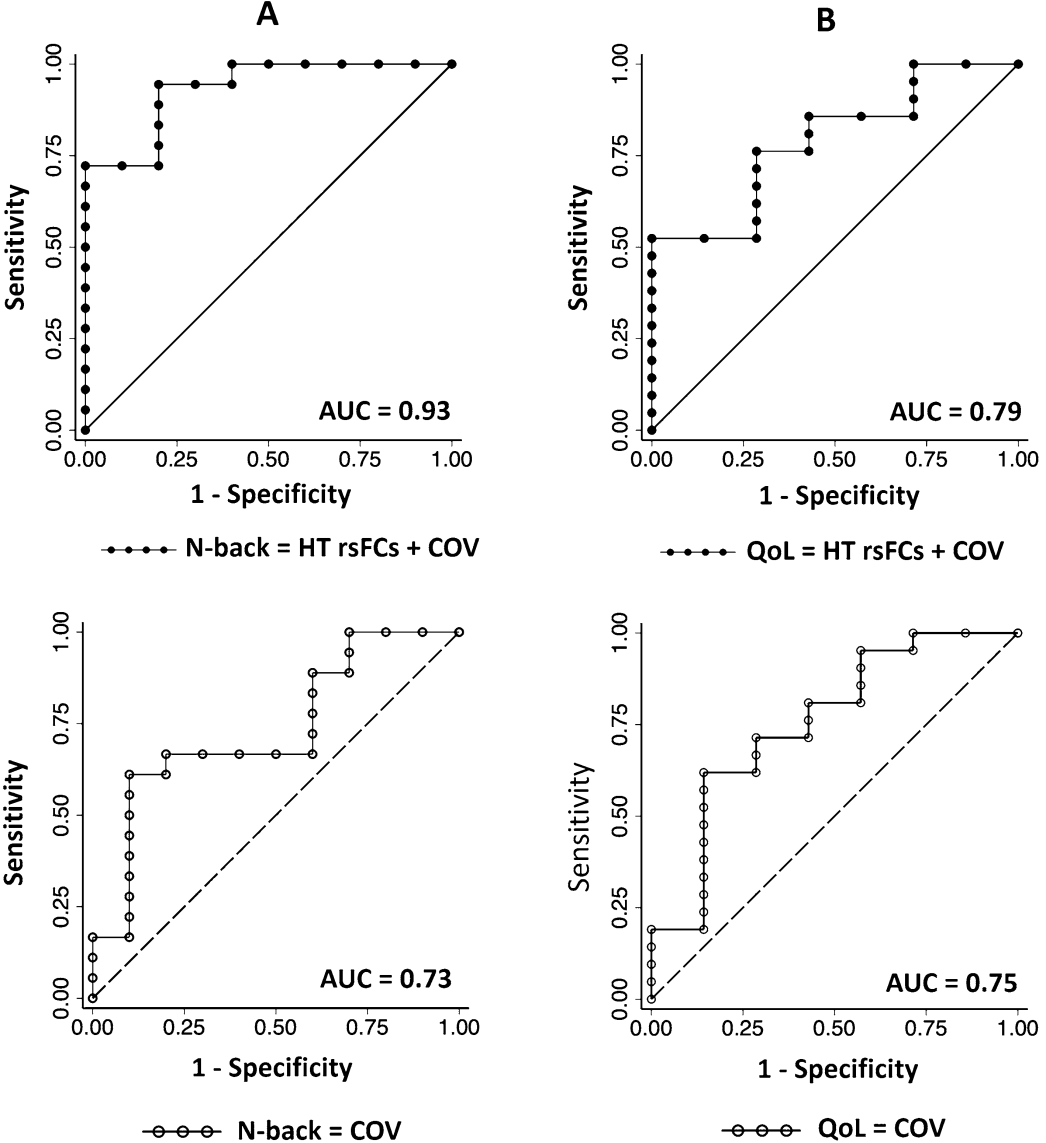
Receiver operating characteristic (ROC) analysis showing the accuracy (area under the curve, AUC) of baseline hypothalamus (HT) rsFC and covariates (solid line and circles) and covariates alone (dashed lines and open circles) in predicting overall changes (follow-up vs. baseline) in (**A**) N-back metrics (as revealed in latent class analysis) and (**B**) QoL score (total QoL score). Covariates (COV) included baseline age, education, and MoCA score.

Additionally, with LCA classes of N-back performance as the response variable (see “Methods”), the logistic regression model with the hypothalamus rsFCs (as noted in previous section) as predictors and baseline age, education, and MoCA scores as covariates was significant (p = 0.010) with an AUC = 0.93 (Fig. 4A). In contrast, the regression model with the covariates alone was not significant (p = 0.119) and showed an AUC = 0.73.

For overall QoL, the logistic regression models with hypothalamus-ACC and cuneus rsFC predictiors along with covariates, as well as the model with covariates alone were not significant (p’s > 0.151), each with an AUC = 0.79 and 0.75, respectively (Fig. 4B).

## Discussion

ADT is widely used for the treatment of prostate cancer. ADT offers survival benefits but the associated adverse effects, especially cognitive dysfunction, are not fully understood; and individuals demonstrated significant variation in ADT-elicited cognitive impairment^6^. Despite extensive research on the neural processes underlying the cognitive impairment, there has been no studies aiming specifically to identify neural predictors of individual variation in the impairment. In the present study, we showed that baseline hypothalamus rsFCs may predict individual variation in changes in working memory and QoL following six months of ADT.

ADT for six months did not appear to affect N-back performance and QoL differently from patients who did not receive ADT. ADT for a longer duration may cause greater cognitive changes^1^, although studies have reported no effects on working memory after 12^61^ and 36^43^ months of treatment. Similarly, a meta-analysis noted no adverse effects of ADT on working memory^42^. Thus, the present findings are consistent with this literature, including our earlier studies^2,41^. However, despite a lack of effects on behavioral measures, we showed earlier significant differences in brain activity and functional connectivity. Notably, our previous studies implicated middle frontal cortical activation^2^ and hypothalamus-middle frontal cortical functional connectivity^41^ in ADT N-back performance.

Despite the absence of an overall effect, individuals demonstrated variations in cognitive performance at follow-up vs. baseline. Of the 6 N-back scores, 17 among 28 ADT patients demonstrated impairment in ≥ 2 scores, while a smaller number (n = 6) performed better at follow-up vs. baseline in all 6 scores. Such inter-subject variation in cognitive performance has been noted in women with breast cancer on chemotherapy^44,45^. In a longitudinal assessment, a sub-population (~ 60%) of breast cancer patients experienced subtle chemotherapy-linked cognitive decline^62^ along with altered frontal-cerebellar rsFC in comparison to those who did not undergo chemotherapy or healthy controls^47^. Decrement in frontal cortical gray matter density mediated inter-subject variation in chemotherapy dosage-related impairment in verbal fluency^46^. Here, we demonstrated that hypothalamus connectivity may predict such variations in working memory and QoL in individuals undergoing ADT, as discussed in more detail below.

Studies have also documented the effects of ADT on patients’ quality of life^63–65^. We assessed different domains of QoL using FACT-P questionnaire and did not observe significant changes in QoL, except possibly PWB, which was marginally reduced (p = 0.078) during follow-up as compared to baseline in ADT. These findings appear to accord with an earlier study which noted worsening physical but comparable emotional and social well-being in prostate cancer patients with 24 weeks of ADT, relative to controls^64^.

Hypothalamus regulates the activity of the HPA axis^66^. Although best known for its role in supporting survival-related responses, the hypothalamus connects structurally with many brain regions, including the amygdala, cingulate and other frontal cortical regions^67^, suggesting that it may partake in cognitive and affective processes beyond those to meet immediate physiological needs. We assessed hypothalamus rsFCs in predicting inter-subject variations in N-back performance and QoL score/sub-scores post-ADT and observed both positive and negative associations. Higher baseline hypothalamus-frontal cortical rsFC predicted worse N-back score. Although we could not explain the precise mechanism of this association, hypothalamus-frontal cortical rsFC is implicated in cognitive and attention control both in health^68^ and illnesses^9,31,69,70^. In our earlier studies too, we noted significant ADT-associated alterations in frontal cortical activation and connectivity^2,41^. We also observed that lower baseline hypothalamus-SPL rsFC predicted longer reaction time and thus, worse attention, in the N-back task, consistent with SPL being part of dorsal attention network^71^. Hypothalamus-ACC and cuneus rsFC each predicted better and worse physical well-being. A hub of the salience network, the ACC connects with both limbic and prefrontal cortex^72^. With the hypothalamus, it regulates autonomic^72^ and visceromotor functions^73^, and hypothalamus-ACC connectivity has been implicated in reward^74^ and pain^75^ processing. The functional significance of hypothalamus-cuneus connectivity is not clear, but may involve visual processing of emotionally relevant stimuli^76,77^. Notably, the associations were not significant in CON, possibly indicating the specificity of hypothalamic rsFCs for ADT-associated changes.

Prediction analyses revealed reasonable accuracy of hypothalamus rsFCs in determining the N-back performance and QoL. The best model constituted hypothalamus-frontoparietal rsFC in predicting N-back performance and hypothalamus-cingulate/occipital rsFC in predicting QoL. Thus, prognostic functional connectivity markers may prove of clinical utility as the acquisition and analysis of functional brain imaging data is easy to standardize, less demanding to the patients, and less prone to subjective errors.

## Limitations and conclusions

A number of limitations need to be considered. First, the study comprised a small sample and the results should be replicated in a larger population. On the other hand, we wish to emphasize that the imaging results on hypothalamic rsFCs were obtained at a corrected threshold and would likely be robust. Second, studies are also needed to employ a more comprehensive battery of neuropsychological tests to fully investigate potential cognitive dysfunction in prostate cancer patients receiving ADT. It is possible that patients may compensate functionally in certain but not in other cognitive domains. Thus, a detailed assessment would reveal the cognitive side effects of ADT. Third, although we targeted the hypothalamus on the basis of the biological effects of androgen deprivation, other brain regions, e.g., frontal cortex, should be thoroughly investigated in future studies. Fourth, as patients may undergo ADT for a longer duration, the current findings should be considered as specific to patients with only 6 months of androgen deprivation. Finally, as we did not observe significant changes in N-back/QoL scores at follow-up compared to baseline in ADT with respect to HC, we cannot entirely rule out the effect of time in driving the changes at follow-up. Nonetheless, in predicting the changes we showed the hypothalamus rsFC associations to be significant in ADT but not in CON, suggesting that these findings may be specific to androgen deprivation.

To conclude, androgen deprivation for 6 months did not lead to significant changes in working memory or quality of life. However, individuals varied in the changes in working memory and quality of life likely as a manifestation of the effects of ADT and indirect effects in functional compensation. Baseline hypothalamus-frontoparietal and occipital/limbic rsFCs predict inter-subject variations in working-memory and QoL in prostate cancer patients at six months after ADT. Studies with longer-term ADT and other cognitive/behavioral markers are needed to fully evaluate the effects of androgen deprivation on cognition.

## Supplementary Information

Supplementary Information.

## Data Availability

The datasets generated and/or analyzed in the paper are part of an on-going study and thus not publicly available. However, the data would be available from the corresponding author on reasonable request.

## Abbreviations

ADT: Androgen deprivation therapy
ACC: Anterior cingulate cortex
AUC: Area under the curve
CON: Controls
EWB: Emotional well-being
FACT-P: Functional Assessment of Cancer Therapy-Prostate
FWB: Functional well-being
HPG: Hypothalamus–pituitary–gonadal
LCA: Latent class analysis
MFG: Middle frontal gyrus
MoCA: Montreal cognitive assessment
MRI: Magnetic resonance imaging
PCS: Prostate cancer subscale
PCG: Precentral gyrus
PWB: Physical well-being
QoL: Quality of life
rsFC: Resting-state functional connectivity
SPL: Superior parietal lobule
SWB: Social well-being
