# Supplementary material for: Hypothalamic connectivities predict individual differences in ADT-elicited changes in working memory and quality of life in prostate cancer patients
Source: Sci Rep. 2022 Jun 10;12:9567. doi: 10.1038/s41598-022-13361-4 (PMC9187668; doi:10.1038/s41598-022-13361-4)
Supplement: Supplementary file 1 — Supplementary Information. [file 41598_2022_13361_MOESM1_ESM.docx]

**Chaudhary et al., Hypothalamic connectivities predict individual differences in ADT-elicited changes in working memory and quality of life in prostate cancer patients.**

**Supplementary Materials**

**Methods**


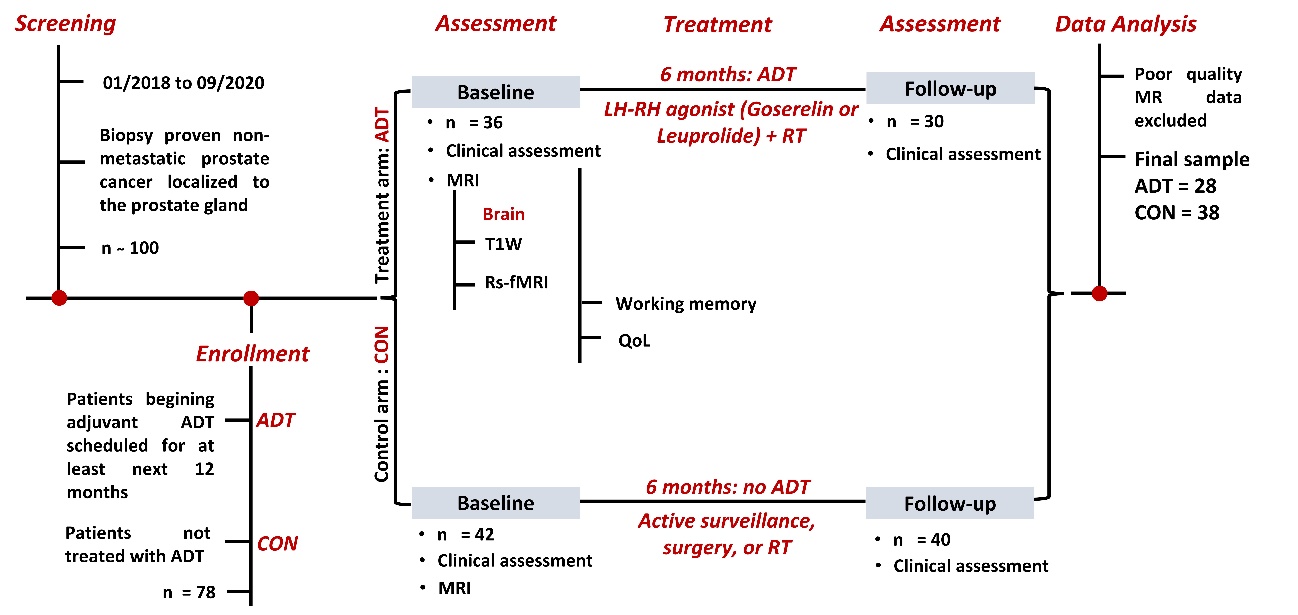


**Supplementary Figure S1.** Study timeline. Note: Treatment for the patients followed current guidelines and are independent of the current study. Three patients of the ADT group had previously undergone surgery. ADT: androgen deprivation therapy, CON: control, MRI: magnetic resonance imaging, T1W: T1 weighted imaging, rs-fMRI: resting state functional MRI, QoL: quality of life, LH-RH: luteinizing hormone releasing hormone, RT: radiation therapy


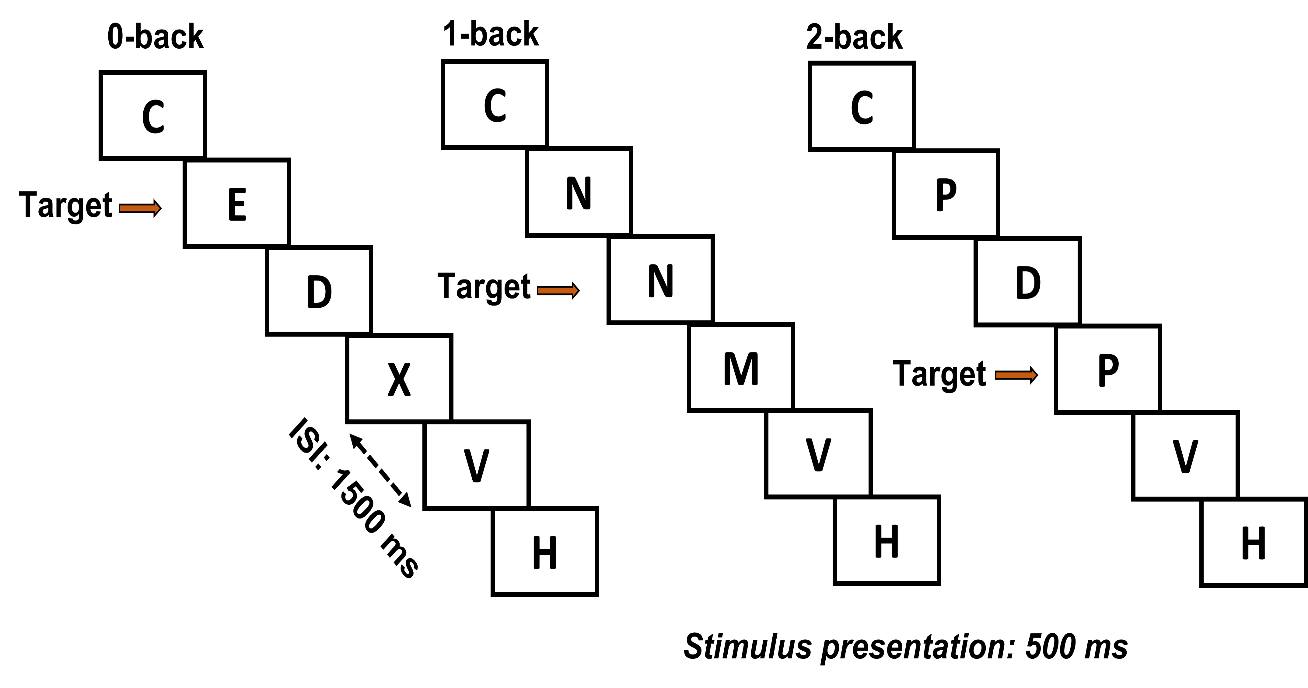


**Figure S2**. N-back working memory task. A stream of fifteen phonologically distinct letters appears in sequency each for a duration of 500 ms and with an inter-stimulus-interval of 1500 ms. There are three different conditions: 0-, 1-, and 2- back, differing in working memory load. In the 0-back trials, participants identified a pre-specified target (e.g., letter “E”); in the 1- and 2- back trials, there is no fixed target; in contrast, a letter that is the same as the one 1- and 2-time steps back represents the target, respectively. Participants were instructed to response as accurately and as fast as possible. N-back task was administered at baseline and 6-month follow-up outside the scanner. Each subject completed 3 sessions of the task, with each session containing two each of 0-, 1-, and 2- back blocks, the order of which was counter-balanced across sessions. Each block began with an information screen showing the ‘‘working memory load’’ for that block (5 s) and contained 24 trials, with one-third showing a target. Correct response rate and reaction time, averaged across blocks and sessions, each for 0-, 1- and 2- back trials, serves as an outcome measure of N-back performance.

**Imaging Protocol**

Subjects were scanned at baseline. Brain imaging was performed on a 3-Tesla Siemens Trio TIM system equipped with a 32-channel head coil. Scanning used the conventional T1-weighted spin echo sagittal anatomical images for slice localization. Next, anatomical images of the functional slice localization were acquired using spin echo imaging in the axial plane parallel to the AC–PC line with TR = 1900ms, TE = 2.52ms, bandwidth = 170 Hz/pixel, flip angle = 9°, field of view = 250 × 250 mm, matrix = 256 × 250 mm, 176 slices with slice thickness = 1 mm and no gap. Resting state functional, blood oxygen level-dependent (BOLD) signals with participants in eyes-closed condition, were then acquired with a single-shot gradient echo echoplanar imaging (EPI) sequence in 51 axial slices parallel to the AC–PC line covering the whole brain using TR = 1,000ms, TE = 30ms, bandwidth = 2290 Hz/pixel, flip angle = 62°, field of view = 210 × 210 mm, matrix = 84 × 84, slice thickness = 2.5 mm without gap, and total acquisition time = 10 m.

**Imaging Data Analysis**

**Resting state functional connectivity**

Data were analyzed with Statistical Parametric Mapping (SPM12). Standard preprocessing pipeline was applied on functional images after first discarding the images corresponding to initial five TRs to achieve signal steady state equilibrium. Next, the functional images of each subject were slice time corrected to correct for the temporal offset between slices, and then were motion corrected (realigned). Realignment produced a mean functional image which was co-registered with high resolution structural image and segmented for normalization with affine registration followed by nonlinear transformation. The estimated normalization parameters were subsequently applied to the corresponding functional image volumes for each subject. Next, the functional images were normalized to Montreal Neurological Institute (MNI) space with resampled voxel size of (2.5×2.5×2.5) mm^3^ and then, were smoothed with a Gaussian kernel of 6-mm FWHM.

Nuisance signals unlikely to reflect neural activity, were removed using linear regression by including the six motion parameters from realignment, signal from whole brain, ventricular system, white matter, and their first-order derivative ^1–5^. Next, functional images were checked for micro-head motion (>0.1 mm) as this may lead to spurious correlations in rsFC analysis, followed by “scrubbing” to remove time points affected by head motions, as successfully applied in previous studies using the thresholds of FD(t)>0.5 mm ^1,6^ or DVARS(t) >75 ^1,7^. Before computing the correlation maps to estimate rsFC, we applied a temporal band-pass filter (0.009 Hz<f<0.08 Hz) to the time course to obtain low-frequency fluctuations ^3–5,8,9^.

We employed the hypothalamus mask from the WFU Pick-Atlas ^10^ as the seed, according to previous study ^11^. The correlation coefficients between the averaged time course of the hypothalamus seed and time courses of all other brain voxels were computed for each participant. Next, the correlation maps were converted into z-score maps by Fisher’s Z transform: z = 0.5log_e_ [1+r/1-r] (r=correlation coefficient) to get normally distributed correlation map.

These maps were further used for group-level analysis as explained in main text.

**Results**

**Table S1.** Treatment (ADT vs. CON) and time (baseline vs. follow-up) main and interaction effects of N-back performance and QoL scores: mixed model analysis

|  | Treatment  (*F_1, 64,_ p*) | Time  (*F_1, 64,_ p*) | Treatment × time  (*F_1, 64,_ p*) |
| --- | --- | --- | --- |
| *N-back correct response %* | | | |
| 0-back | 4.18, 0.041* | 3.72, 0.054 | 0.50, 0.478 |
| 1-back | 6.16, 0.013* | 0.80, 0.370 | 2.69, 0.101 |
| 2-back | 0.83, 0.364 | 3.96, 0.046* | 1.65, 0.199 |
| *N-back correct trial RT (ms)* | | | |
| 0-back | 1.08, 0.299 | 0.02, 0.899 | 0.47, 0.494 |
| 1-back | 0.13, 0.720 | 0.32, 0.571 | 0.56, 0.456 |
| 2-back | 1.51, 0.219 | <0.001, 0.954 | 0.01, 0.939 |
| *Quality of life scores* | | | |
| Total | 5.98, 0.015* | 0.02, 0.899 | 0.08, 0.778 |
| PWB | 6.85, 0.008* | 5.40, 0.020* | 0.97, 0.329 |
| SWB | 1.78, 0.182 | 0.24, 0.624 | 0.28, 0.598 |
| EWB | 3.24, 0.072 | 2.77, 0.096 | 1.28, 0.257 |
| FWB | 3.43, 0.064 | 0.09, 0.759 | 0.51, 0.477 |
| PCS | 5.86, 0.016* | 2.24, 0.135 | 0.52, 0.472 |

Note: *p<0.05; PWB: Physical well-being, SWB: Subjective well-being, EWB: emotional well-being, FWB: functional well-being, PCS: prostate-cancer specific**.**

**Table S2.** Participants’ N-back task performance and QoL scores at baseline and 6-month follow-up

|  | ADT_B | ADT_F | CON_B | CON_F |
| --- | --- | --- | --- | --- |
| *correct response rate (%)* | | | | |
| 0-back | 97.5 ± 5.2 | 96.7 ± 5.2 | 99.6 ± 1.3 | 97.8 ± 4.3 |
| 1-back | 85.3 ± 11.7 | 79.2 ± 22.4 | 89.0 ± 13.8 | 90.8 ± 14.4 |
| 2-back | 65.6 ± 18.5 | 58.4 ± 17.9 | 66.7 ± 20.9 | 65.1 ± 18.9 |
| *Reaction time of correct trials (ms)* | | | | |
| 0-back | 533.4 ± 101.9 | 541.0 ± 63.2 | 523.9 ± 117.8 | 512.7 ± 69.1 |
| 1-back | 641.6 ± 122.7 | 663.2 ± 109.0 | 643.3 ± 166.4 | 640.3 ± 130.5 |
| 2-back | 778.2 ± 187.5 | 775.1 ± 192.2 | 730.8 ± 191.6 | 731.3 ± 133.6 |
|  |  |  |  |  |
| Quality of life | 113 ± 20 | 112 ± 18 | 122 ± 18 | 122 ± 17 |
| PWB | 23.8 ± 3.8 | 22.3 ± 3.9 | 25.3 ± 2.9 | 24.7 ± 3.0 |
| SWB | 20.1 ± 7.0 | 20.8 ± 4.1 | 21.9 ± 4.8 | 21.9 ± 4.3 |
| EWB | 19.0 ± 3.9 | 20.1 ± 3.3 | 20.7 ± 3.2 | 20.9 ± 2.6 |
| FWB | 17.7 ± 8.6 | 18.7 ± 5.7 | 20.7 ± 5.7 | 20.3 ± 4.8 |
| PCS | 32.2 ± 7.5 | 30.3 ± 6.5 | 35.4 ± 7.7 | 34.7 ± 6.5 |

Note: Values are mean ± SD; B: baseline, F: follow-up

**Table S3.** Memory sub-processes at baseline and 6-month follow-up

|  | ADT_B | CON_B | p | ADT_F | CON_F | p |
| --- | --- | --- | --- | --- | --- | --- |
| *correct response rate (%)* | | | | | | |
| Load | -31.9±18.1 | -32.9±20.85 | 0.831 | -38.3±17.4 | -32.7±18.3 | 0.217 |
| Replacement | -12.1±11.3 | -10.6±13.7 | 0.626 | -17.5±22.2 | -6.9±13.6 | 0.020* |
| Shift | -19.7±14.0 | -22.3±18.6 | 0.537 | -20.7±22.6 | -25.7±15.6 | 0.295 |
| *Reaction time of correct trials (ms)* | | | | | | |
| Load | 245±153 | 207±121 | 0.267 | 234±169 | 218±112 | 0.658 |
| Replacement | 108±84 | 119±98 | 0.630 | 122±102 | 127±98 | 0.828 |
| Shift | 136±120 | 88±128 | 0.119 | 112±163 | 91±98 | 0.519 |

Note: Data in mean ± SD; B: baseline, F: follow-up; *p-value <0.05, two-sample t-test of ADT vs. CON. No measures showed significant differences between follow-up and baseline as assessed by paired t-test (p’s >0.05) in ADT or CON. Maintenace load: 2-back minus 0-back; replacement: 1-back minus 0-back; and shift: 2-back minus 1-back ^12^.


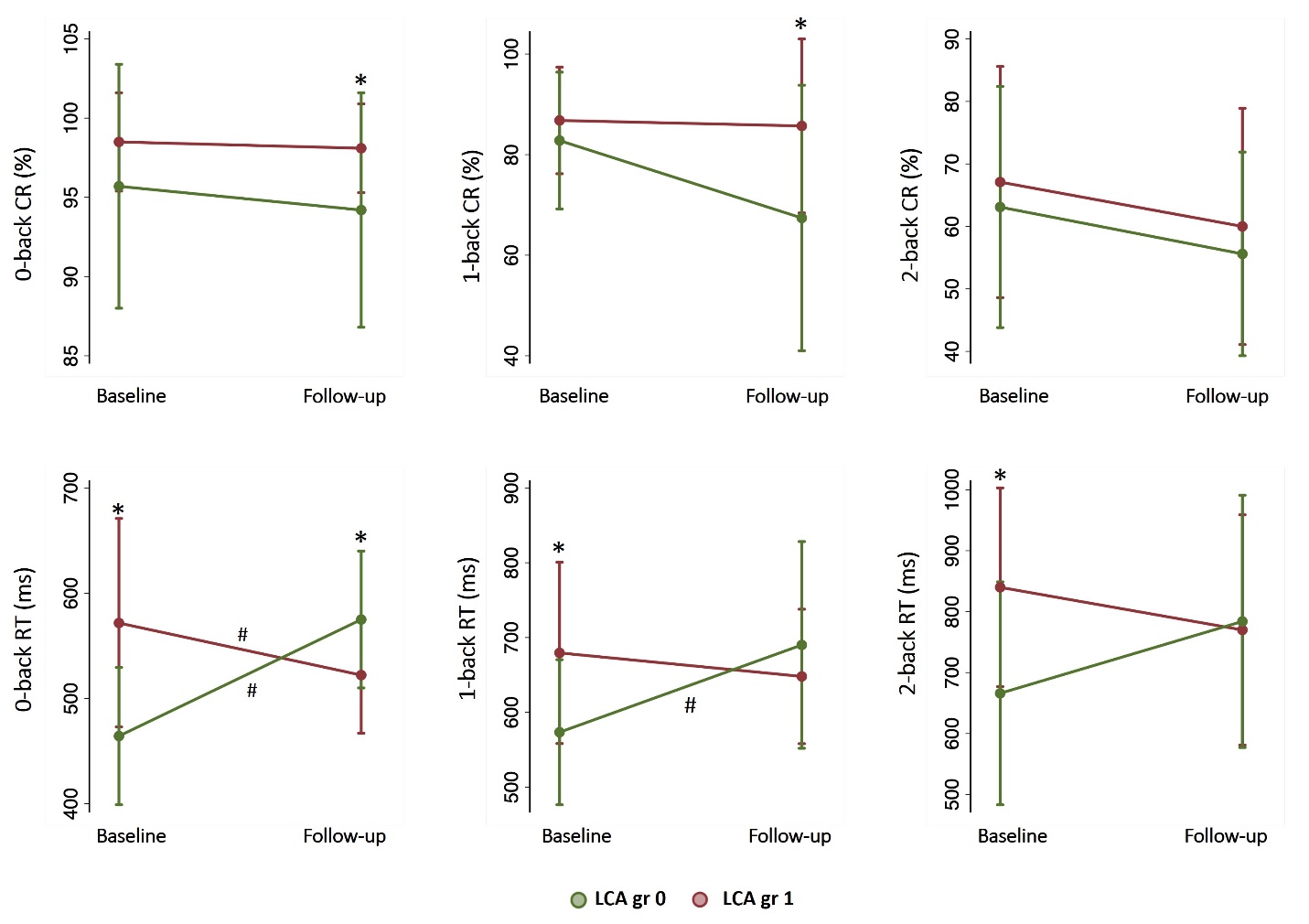


**Figure S3.** Distribution of N-back correct response (CR) rates (%), and N-back response time (RT, ms) at baseline and follow-up in ADT LCA group 0 (green, n= 10) and group 1 (red, n= 18). Data shown in group mean ± SD; LCA: latent class analysis, *p<0.05 (two-sample t-test of the two groups); #p<0.05 (paired t-test); 1-/2-back CR and RT follow-up vs. baseline changes in LCA group 0 were marginally significant (0.07<p’s<0.10).


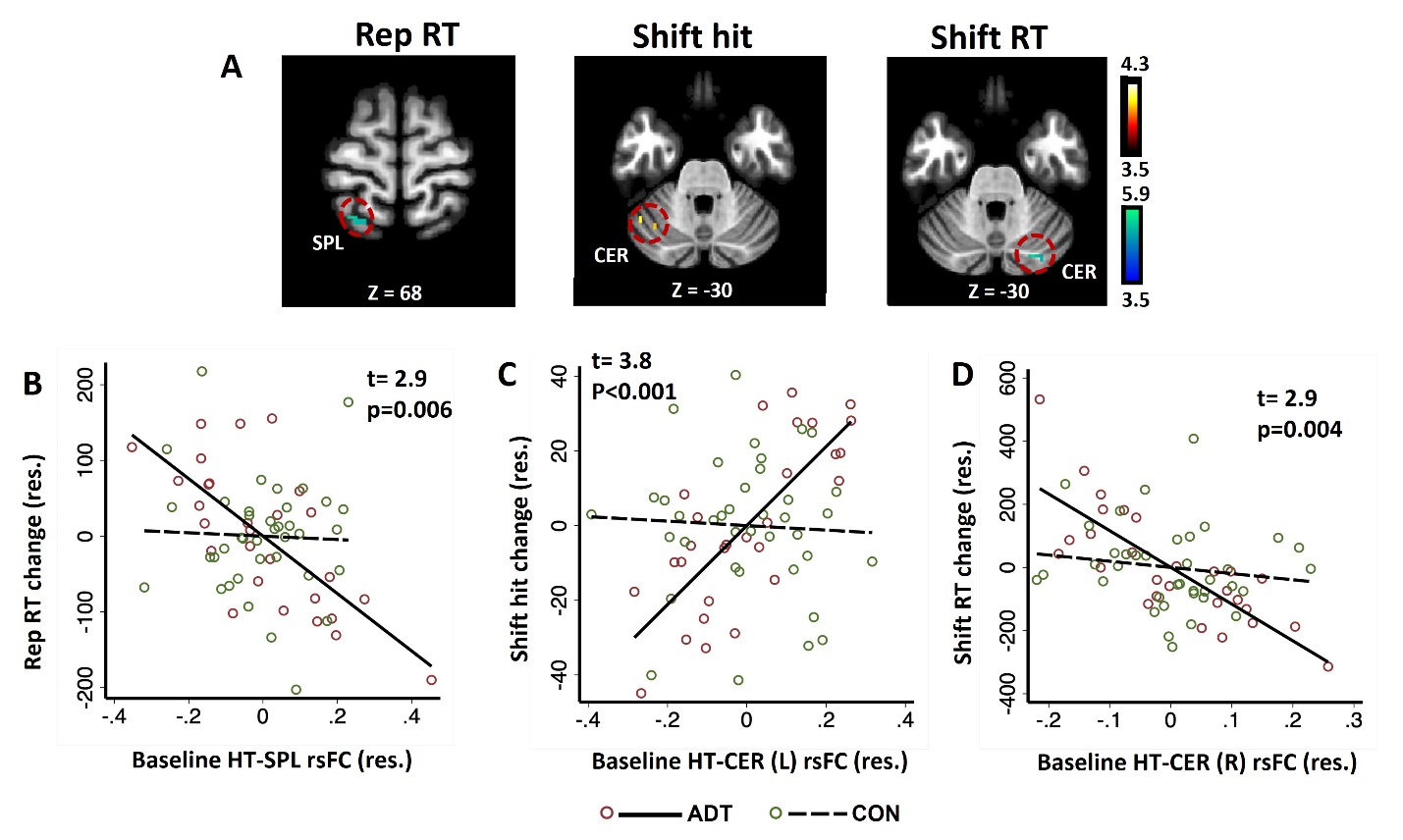


**Figure S4.** Hypothalamic (HT) rsFC correlates of changes in subprocesses of N-back memory in ADT as assessed using whole-brain regression with baseline age, education, MoCA as covariates. Clusters were identified with the same threshold: cluster p<0.05 FWE-corrected and cluster-forming voxel p<0.001, uncorrected. (**A**) Baseline HT rsFC predictors of change (follow-up versus baseline) in N-back sub-scores: Replacement (Rep) reaction time (RT) (1-back RT minus 0-back RT), Shift correct response rate (hit) (2-back hit minus 1-back hit), and Shift RT (2-back RT minus 1-back RT). (B) left superior parietal lobule (SPL) rsFC and change in replacement (Rep) RT, (C) left cerebellum (CER) rsFC and change in shift hit rate, and (D) right CER rsFC and change in shift RT. Color bar shows voxel T-values. Warm/cool colors: positive/negative correlations. Note that, because of the inclusion of covariates in the regression, the residuals are plotted here. The insets show the t- and p-values of slope tests of ADT vs. CON in the regressions.

**Additional Discussion**

The replacement correct response rate was lower in ADT as compared to CON. We also observed that lower baseline hypothalamus-SPL rsFC predicted longer reaction time and thus, worse attention, during memory replacement, consistent with SPL being part of dorsal attention network ^13^. Higher baseline hypothalamus-cerebellum rsFC predicted better memory shift, in accord with cerebellar participation in working memory ^14,15^, especially in storage and replacement of memory ^16^. Notably, the associations were not significant in CON, possibly indicating the specificity of hypothalamic rsFCs for ADT-associated changes in N-back subprocesses.

**Supplementary References:**

1. Zhang, S., Wang, W., Zhornitsky, S. & Li, C.-S. R. Resting State Functional Connectivity of the Lateral and Medial Hypothalamus in Cocaine Dependence: An Exploratory Study. *Front. psychiatry* **9**, 344 (2018).

2. Rombouts, S. A. R. B., Stam, C. J., Kuijer, J. P. A., Scheltens, P. & Barkhof, F. Identifying confounds to increase specificity during a ‘no task condition’. Evidence for hippocampal connectivity using fMRI. *Neuroimage* **20**, 1236–1245 (2003).

3. Fox, M. D. *et al.* The human brain is intrinsically organized into dynamic, anticorrelated functional networks. *Proc. Natl. Acad. Sci. U. S. A.* **102**, 9673–9678 (2005).

4. Fair, D. A. *et al.* A method for using blocked and event-related fMRI data to study ‘resting state’ functional connectivity. *Neuroimage* **35**, 396–405 (2007).

5. Fox, M. D. & Raichle, M. E. Spontaneous fluctuations in brain activity observed with functional magnetic resonance imaging. *Nat. Rev. Neurosci.* **8**, 700–711 (2007).

6. Power, J. D., Barnes, K. A., Snyder, A. Z., Schlaggar, B. L. & Petersen, S. E. Spurious but systematic correlations in functional connectivity MRI networks arise from subject motion. *Neuroimage* **59**, 2142–2154 (2012).

7. Li, J. *et al.* Global signal regression strengthens association between resting-state functional connectivity and behavior. *Neuroimage* **196**, 126–141 (2019).

8. Cordes, D. *et al.* Frequencies contributing to functional connectivity in the cerebral cortex in ‘resting-state’ data. *AJNR. Am. J. Neuroradiol.* **22**, 1326–1333 (2001).

9. Lowe, M. J., Mock, B. J. & Sorenson, J. A. Functional connectivity in single and multislice echoplanar imaging using resting-state fluctuations. *Neuroimage* **7**, 119–132 (1998).

10. Maldjian, J. A., Laurienti, P. J., Kraft, R. A. & Burdette, J. H. An automated method for neuroanatomic and cytoarchitectonic atlas-based interrogation of fMRI data sets. *Neuroimage* **19**, 1233–1239 (2003).

11. Le, T. M. *et al.* The interrelationship of body mass index with gray matter volume and resting-state functional connectivity of the hypothalamus. *Int. J. Obes.* **44**, 1097–1107 (2020).

12. Chen, Y.-N., Mitra, S. & Schlaghecken, F. Sub-processes of working memory in the N-back task: an investigation using ERPs. *Clin. Neurophysiol.* **119**, 1546–59 (2008).

13. Petersen, S. E. & Posner, M. I. The attention system of the human brain: 20 years after. *Annu. Rev. Neurosci.* **35**, 73–89 (2012).

14. Ziemus, B. *et al.* Impaired working-memory after cerebellar infarcts paralleled by changes in BOLD signal of a cortico-cerebellar circuit. *Neuropsychologia* **45**, 2016–24 (2007).

15. Liu, Q., Liu, C., Chen, Y. & Zhang, Y. Cognitive Dysfunction following Cerebellar Stroke: Insights Gained from Neuropsychological and Neuroimaging Research. *Neural Plast.* **2022**, 3148739 (2022).

16. Marvel, C. L. & Desmond, J. E. Chapter 3 - The Cerebellum and Verbal Working Memory. in (eds. Mariën, P. & Manto, M. B. T.-T. L. C.) 51–62 (Academic Press, 2016). doi:https://doi.org/10.1016/B978-0-12-801608-4.00003-7.
